# A survey tool for measuring evidence-based decision making capacity in public health agencies

**DOI:** 10.1186/1472-6963-12-57

**Published:** 2012-03-09

**Authors:** Julie A Jacobs, Paula F Clayton, Cassandra Dove, Tanya Funchess, Ellen Jones, Ghazala Perveen, Brandon Skidmore, Victor Sutton, Sarah Worthington, Elizabeth A Baker, Anjali D Deshpande, Ross C Brownson

**Affiliations:** 1 Prevention Research Center in St. Louis, Brown School, Washington University in St. Louis, St. Louis, MO, USA; 2 Bureau of Health Promotion, Kansas Department of Health and Environment, Topeka, KS, USA; 3 Office of Preventive Health, Mississippi State Department of Health, Jackson, MS, USA; 4 Office of Tobacco Control, Mississippi State Department of Health, Jackson, MS, USA; 5 School of Health Related Professions, University of Mississippi Medical Center, and National Association of Chronic Disease Directors, Jackson, MS, USA; 6 Active Living KC, Kansas City Health Department, Kansas City, MO, USA; 7 Prevention Research Center in St. Louis, Saint Louis University School of Public Health, St. Louis, MO, USA; 8 Division of Health Behavior Research, Washington University School of Medicine, Washington University in St. Louis, St. Louis, MO, USA; 9 Division of Public Health Sciences, Alvin J. Siteman Cancer Center, Washington University School of Medicine, St. Louis, MO, USA; 10 George Warren Brown School of Social Work, Division of Public Health Sciences, School of Medicine, Washington University in St. Louis, Kingshighway Building 660 S. Euclid Campus, Box 8109, St. Louis, MO 63110, USA

**Keywords:** Evidence-based practice, Public health

## Abstract

**Background:**

While increasing attention is placed on using evidence-based decision making (EBDM) to improve public health, there is little research assessing the current EBDM capacity of the public health workforce. Public health agencies serve a wide range of populations with varying levels of resources. Our survey tool allows an individual agency to collect data that reflects its unique workforce.

**Methods:**

Health department leaders and academic researchers collaboratively developed and conducted cross-sectional surveys in Kansas and Mississippi (USA) to assess EBDM capacity. Surveys were delivered to state- and local-level practitioners and community partners working in chronic disease control and prevention. The core component of the surveys was adopted from a previously tested instrument and measured gaps (importance versus availability) in competencies for EBDM in chronic disease. Other survey questions addressed expectations and incentives for using EBDM, self-efficacy in three EBDM skills, and estimates of EBDM within the agency.

**Results:**

In both states, participants identified communication with policymakers, use of economic evaluation, and translation of research to practice as top competency gaps. Self-efficacy in developing evidence-based chronic disease control programs was lower than in finding or using data. Public health practitioners estimated that approximately two-thirds of programs in their agency were evidence-based. Mississippi participants indicated that health department leaders' expectations for the use of EBDM was approximately twice that of co-workers' expectations and that the use of EBDM could be increased with training and leadership prioritization.

**Conclusions:**

The assessment of EBDM capacity in Kansas and Mississippi built upon previous nationwide findings to identify top gaps in core competencies for EBDM in chronic disease and to estimate a percentage of programs in U.S. health departments that are evidence-based. The survey can serve as a valuable tool for other health departments and non-governmental organizations to assess EBDM capacity within their own workforce and to assist in the identification of approaches that will enhance the uptake of EBDM processes in public health programming and policymaking. Localized survey findings can provide direction for focusing workforce training programs and can indicate the types of incentives and policies that could affect the culture of EBDM in the workplace.

## Background

Chronic diseases, such as heart disease, cancer and diabetes, are responsible for about 60% of all deaths globally and 70% of deaths in the United States [1,2] with morbidity and mortality projected to increase both nationally and internationally over the next several decades [1,3]. Physical inactivity, poor diet, tobacco use, alcohol consumption, and other modifiable behavioral risk factors account for a substantial number of these deaths [4,5], allowing ample intervention opportunities through public health programs and policies.

Calls for the use of evidence-based decision making (EBDM) processes to develop chronic disease control and prevention programs come from both academia and practice, including major health organizations such as the World Health Organization and the Centers for Disease Control and Prevention [6-10]. The concept of EBDM in public health has evolved over the past decade and can be summarized as a process that utilizes the best available scientific evidence regarding the effectiveness of various programs or policies and translates that evidence to real world practice by incorporating community-level data, resources, and priorities [11,12].

There is a well-recognized gap between the production of scientific evidence and the use of that evidence in "real world" settings [13-16] (e.g., policy making bodies, health departments). Closing the translation gap is a complicated process, and increasing amounts of literature address this topic, often referred to as "knowledge transfer" or "dissemination and implementation research" [13-16]. The use of EBDM in public health agencies depends on many factors, including the training and experience of the workforce, organizational resources and climate (e.g., funding, buy-in from leadership and elected officials), and the availability, applicability, and dissemination of evidence on a given topic [16-21].

Research is needed to understand the determinants and approaches that will enhance the uptake of EBDM processes in public health agencies. We conducted a two phase research project that aimed to increase the use of chronic disease evidence-based interventions (EBIs) in public health agency settings. In the first phase, 447 state-level chronic disease practitioners across the U.S. completed a survey that assessed the importance, availability, and use of various components of EBDM in chronic disease. Through quantitative and qualitative methods, we examined practitioner's barriers and solutions to improving the use of EBIs in state health departments and assessed gaps in the importance and availability of core chronic disease competencies [22-24].

In phase 2, we conducted in-depth projects in two U.S. states: Kansas and Mississippi. Under the U.S. constitutional doctrine of reserved powers, the states retain enormous authority to protect the public's health [25]. The states shoulder their broad public health responsibilities through work carried out by state and local health agencies. These interventions are primarily focused on chronic disease prevention and control (primary and secondary prevention), not on management of chronic disease. Non-governmental organizations (NGOs) and other community partners also play critical roles in public health, providing health services and implementing interventions and policy changes in a variety of capacities.

There are large variations in the populations these agencies serve, their types of governance, the services they provide, and the education and job functions of their staff [26-30]. Due to this heterogeneity in public health agencies, it is important to have tools to collect localized data that reflect the unique nature of an agency's workforce and community partners [31]. We began phase 2 by developing a brief survey tool to assess baseline capacity for EBDM, seeking to identify specific targets for increasing the dissemination of EBIs in these two states. This article presents methods and findings from the initial phase 2 survey assessment with the goal of encouraging other public health agencies, in the U.S. and across the globe, to assess EBDM in their own workforce.

## Methods

### State selection

Kansas and Mississippi were chosen for this study based on their recent completion of a State Technical Assistance and Review (STAR) Program through the National Association of Chronic Disease Directors (NACDD) [32]. Seven states had completed the STAR program at the time of selection (September 2009), but for feasibility and resource reasons, only two were chosen for this study. The STAR process involved self-study by the state along with a four-day site visit by an experienced chronic disease control and prevention team. Prior to involvement in this research project, both Kansas and Mississippi had identified strengths, challenges and priorities of their chronic disease units, and they were beginning to implement recommendations from the STAR report. The STAR program recommends that states conduct ongoing assessment, and the current survey helped fulfill this function for Kansas and Mississippi.

### Kansas survey development

The leader of the Kansas chronic disease unit selected a small team of health department employees to participate in this research project. The Kansas team consisted of the Director and Deputy Director of the Bureau of Health Promotion and the Director of Science and Surveillance/Health Officer II. Through monthly conference calls and email communications, the Kansas team and academic researchers collaboratively developed the survey instrument and sampling plan.

The majority of this cross-sectional survey was derived from the 74-question national survey used in the first phase of our study [22-24]. The content of that national survey was informed by previous work regarding a card sorting exercise that rated competencies for evidence-based cancer control [33], and the survey underwent cognitive response testing. The Kansas team customized job-related demographic questions (e.g., job title, program area specialty). Four new survey questions were added. Three addressed the self-efficacy of EBDM skills and one produced an estimate of evidence-based programs within one's agency. The Kansas survey contained 33 questions and was estimated to take less than 15 minutes to complete.

The Kansas team identified employees and partners who worked in chronic disease control and prevention. Kansas survey recipients included state and local health department practitioners as well as academic, coalition and volunteer community partners. In this decentralized state health department (SHD), state officials did not have access to complete contact lists for local health department (LHD) practitioners. Prior to the survey's launch, we contacted LHD directors from the 13 counties that served the largest populations and asked them to identify employees who worked in chronic disease control and prevention. The survey was initially delivered to all LHD directors and to the additional practitioners identified in those 13 counties. Using a snowball sampling technique, we also allowed all LHD survey respondents to identify colleagues who worked in chronic disease. After verifying their employment and excluding any duplicate names, we delivered the survey to those colleagues as well.

### Mississippi survey development

Mississippi survey development followed the same process. The Mississippi team included the Director of the Office of Preventive Health, the Director and Deputy Director of the Chronic Disease Bureau, and an NACDD consultant. Demographic questions were customized and the 4 questions added to the Kansas survey were retained in the Mississippi survey. Due to concerns that respondents would consider topics such as immunizations and infectious disease when answering, the Mississippi survey repeated certain questions to ask first about all programs and then specifically about chronic disease programs.

The Mississippi team added a new question regarding expectations to use EBDM ("who expects you to use EBDM related to public health program planning"). Participants could select boxes for health department leaders, direct supervisor, co-workers, and community partners. The Mississippi survey also added a question asking participants to choose their top 2 incentives for using EBDM in their work from the following list: 1) EBDM is given a high priority by leaders in my organization, 2) positive feedback or encouragement, 3) a performance evaluation that considers the use of EBDM, 4) trainings, and 5) professional recognition. The Mississippi survey contained 38 questions and was also designed to be completed in less than 15 minutes.

Survey recipients were identified by the Mississippi team and included state- and district-level public health practitioners. The Mississippi State Department of Health has a centralized relationship with local health departments, and the state is divided into nine districts that each oversee several county health departments.

### Data collection

Prior to the survey distribution, an email co-written by a health department leader and the principal investigator of our research team explained the survey and its importance to each recipient on our contact list. The survey was delivered using ZipSurvey online survey software [34]. Each participant received a unique link to the survey, and nonrespondents received reminder emails. Because incentives increase response rates [35], we offered a $10 gift card to each participant who completed the survey. The Kansas survey was open for 9 weeks from December 2009 to February 2010, and Mississippi's survey was open for 6 weeks from January to March 2010. The survey instruments are available from the last author and in Additional Files 1 and 2 of this manuscript. This study was approved by the Washington University Human Research Protection Office (HRPO #09-1745).

### Analysis

Respondents who answered only demographic questions were not included in descriptive summaries or in response rates. Bivariate relationships were analyzed using independent samples t-tests or Pearson chi-square tests. For the EBDM competencies (see Additional Files 1 and 2 for descriptions), respondents rated both the importance and the availability of the competencies on a scale of 0 (very unimportant or unavailable) to 10 (very important or available). The survey defined availability as "how available you feel each skill is to you when you need it (either in your own skill set or in others')" while importance was not further defined. We created a gap score by subtracting each availability score from the corresponding importance score and calculated a 95% confidence interval (CI) for each.

## Results

The Kansas survey was delivered to 391 valid email addresses and received 190 responses, yielding a 49% response rate. Survey responses were nearly evenly split among SHD practitioners (36%), LHD practitioners (33%) and community partners (31%) (Table 1). Over half (55%) had more than 10 years of experience in public health, and 49% indicated that they held a master's or doctoral degree.

**Table 1:** Participants in evidence-based decision making capacity surveys in Kansas and Mississippi, USA, 2010

	Kansas *n (%)*	Mississippi *n (%)*
Agency		
State Health Department	69 (36.3)	40 (55.6)
Local/District Health Department	63 (33.2)	32 (44.4)
Community Partners	58 (30.5)	

Most Advanced Degree		
Doctorate or Master's	93 (48.9)	41 (56.9)
Bachelors or Some College	88 (46.3)	31 (43.1)
Missing	9.(4.7)	

Years of Public Health Experience		
< 5 years	38 (20.0)	8 (11.1)
5 to < 10 years	45 (23.7)	12 (16.7)
10+ years	104 (54.7)	52 (72.2)
Missing	3 (1.6)	

Gender		
Female	151 (79.5)	56 (77.8)
Male	38 (20.0)	15 (20.8)
Missing	1 (0.5)	1 (1.4)

The Mississippi survey had a 75% response rate with 72 surveys completed out of the 96 delivered. State practitioners represented 56% of the responses, and the remaining 44% were from district health offices. The majority of respondents (72%) had more than 10 years of public health experience, and over half (56%) held a master's or doctoral degree.

Nearly 80% of respondents were female in both Kansas and Mississippi. Also in both surveys, practitioners at the state level were significantly more likely to hold master's or doctoral degrees than those at the local or district level (Kansas p = 0.03, Mississippi p < 0.01). In Kansas, the largest job categories represented were program managers, administrators or coordinators (48%) and health educators (15%). In Mississippi, over a third of the respondents were nurses (35%) while this group represented less than 7% of Kansas' responses.

In both Kansas and Mississippi, the three biggest gaps between the importance and the availability of competencies necessary for EBDM in chronic disease were: transmitting evidence-based research to policymakers, making decisions based on economic evaluation, and translating evidence-based interventions to "real world" settings (Table 2). In Kansas, mean importance and availability scores were higher for state respondents compared to local respondents, and gaps were larger at the local level than at the state level. Mississippi surveys showed more mixed results, and gap scores were larger at the state level compared to the district level.

**Table 2:** Importance, availability, and gaps in competency ratings^‡^, Kansas and Mississippi, USA, 2010

	Kansas *n = 190*			Mississippi *n = 72*		
**Competency**	**All respondents**	**State health department**	**Local health department**	**All respondents**	**State health department**	**District health department**

**Mean (95% Confidence Interval)**						

*Transmitting Research to Policymakers*						

Importance	8.8 (8.6-9.0)	9.1 (8.8-9.4)	8.4 (8.0-8.8)***	8.7 (8.2-9.1)	9.1 (8.6-9.5)	8.1 (7.4-8.8)**

Availability	5.1 (4.8-5.5)	5.5 (4.9-6.1)	4.3 (3.8-4.9)***	5.3 (4.7-5.9)	5.4 (4.6-6.2)	5.0 (4.1-6.0)

Gap	**3.7 (3.4-4.1)**	**3.6 (3.0-4.2)**	**4.1 (3.4-4.7)**	**3.4 (2.8-4.0)**	**3.6 (2.7-4.6)**	**3.1 (2.2-3.9)**

*Decisions Based on Economic Evaluation*						

Importance	8.5 (8.3-8.7)	8.7 (8.3-9.0)	8.2 (7.8-8.6)	8.8 (8.5-9.2)	9.0 (8.6-9.4)	8.5 (8.0-9.1)

Availability	5.1 (4.8-5.5)	5.4 (4.8-6.0)	4.6 (4.0-5.3)*	5.6 (5.0-6.2)	5.4 (4.5-6.2)	6.0 (5.1-6.9)

Gap	**3.4 (3.1-3.7)**	**3.3 (2.7-3.8)**	**3.6 (3.0-4.2)**	**3.2 (2.5-3.9)**	**3.7 (2.7-4.6)**	2.5 (1.7-3.4)*

*Translating Evidence-Based Interventions*						

Importance	8.7 (8.4-8.9)	9.1 (8.8-9.4)	8.0 (7.4-8.5)***	9.1 (8.8-9.4)	9.4 (9.0-9.7)	8.8 (8.4-9.1)**

Availability	5.5 (5.2-5.9)	6.0 (5.5-6.6)	4.7 (4.1-5.3)***	5.6 (5.0-6.2)	5.8 (4.9-6.6)	5.4 (4.4-6.3)

Gap	**3.1 (2.8-3.5)**	**3.0 (2.4-3.6)**	**3.2 (2.7-3.8)**	**3.5 (2.8-4.1)**	**3.6 (2.7-4.4)**	**3.4 (2.4-4.3)**

*Qualitative Evaluation*						

Importance	8.0 (7.7-8.2)	8.4 (8.1-8.7)	7.3 (6.8-7.8)***	8.6 (8.3-8.9)	8.9 (8.5-9.3)	8.2 (7.7-8.7)**

Availability	5.5 (5.1-5.8)	5.9 (5.4-6.5)	4.4 (3.7-5.0)***	5.8 (5.1-6.4)	6.0 (5.1-6.9)	5.4 (4.6-6.3)

Gap	**2.5 (2.2-2.8)**	**2.5 (2.0-3.0)**	**2.9 (2.4-3.5)**	**2.9 (2.2-3.5)**	**2.9 (2.0-3.8)**	**2.8 (1.9-3.6)**

*Developing an Action Plan for Program/Policy*						

Importance	8.7 (8.4-8.9)	9.0 (8.6-9.3)	8.2 (7.7-8.7)***	9.0 (8.7-9.3)	9.2 (8.8-9.6)	8.7 (8.3-9.2)

Availability	6.2 (5.8-6.6)	6.9 (6.3-7.4)	5.1 (4.4-5.7)***	6.0 (5.3-6.6)	6.2 (5.4-7.0)	5.7 (4.7-6.7)

Gap	**2.5 (2.2-2.8)**	**2.1 (1.6-2.6)**	3.1 (2.5-3.7)**	**3.0 (2.4-3.7)**	**3.0 (2.2-3.9)**	**3.0 (2.1-4.0)**

*Multidisciplinary Partnerships*						

Importance	8.9 (8.7-9.1)	9.1 (8.7-9.4)	8.6 (8.2-9.0)*	8.9 (8.6-9.3)	9.2 (8.9-9.6)	8.5 (7.9-9.2)*

Availability	6.2 (5.8-6.5)	6.8 (6.2-7.3)	5.7 (5.1-6.3)**	6.2 (5.6-6.8)	6.2 (5.3-7.0)	6.2 (5.3-7.1)

Gap	**2.7 (2.4-3.1)**	**2.3 (1.7-2.9)**	**2.9 (2.3-3.5)**	**2.7 (2.2-3.3)**	**3.0 (2.2-3.9)**	**2.3 (1.6-3.1)**

*Evaluation Designs*						

Importance	7.4 (7.1-7.7)	7.9 (7.5-8.2)	6.4 (5.9-7.0)***			

Availability	4.9 (4.6-5.3)	5.4 (4.9-5.9)	3.8 (3.2-4.4)***			

Gap	**2.5 (2.1-2.8)**	**2.5 (2.0-2.9)**	**2.6 (2.1-3.2)**			

*Quantitative Evaluation*						

Importance	8.2 (7.9-8.4)	8.5 (8.2-8.9)	7.4 (6.9-7.9)***	8.2 (7.8-8.6)	8.5 (8.0-9.1)	7.8 (7.0-8.5)*

Availability	5.9 (5.5-6.3)	6.7 (6.2-7.2)	4.4 (3.8-5.1)***	5.8 (5.2-6.4)	6.0 (5.2-6.8)	5.6 (4.6-6.5)

Gap	**2.3 (1.9-2.6)**	**1.8 (1.4-2.3)**	3.0 (2.4-3.5)***	**2.4 (1.8-2.9)**	**2.5 (1.8-3.3)**	**2.2 (1.4-3.0)**

*Prioritizing Health Issues*						

Importance	8.3 (8.0-8.5)	8.6 (8.2-8.9)	7.8 (7.4-8.2)***	8.4 (8.0-8.8)	8.6 (7.9-9.2)	8.2 (7.6-8.7)

Availability	6.1 (5.8-6.4)	6.4 (5.9-6.8)	5.3 (4.7-5.9)**	5.9 (5.3-6.4)	5.7 (4.9-6.4)	6.1 (5.3-6.9)

Gap	**2.2 (1.9-2.5)**	**2.2 (1.8-2.6)**	**2.5 (2.0-3.0)**	**2.5 (2.0-3.1)**	**2.9 (2.2-3.7)**	**2.1 (1.3-2.8)**

‡ Likert scale 0-10 with higher scores indicating greater importance/availability state vs. local/district health departments: * p value ≤ 0.10; ** p value ≤ 0.05; *** p value ≤ 0.01

Across all four categories, the percentage of Mississippi respondents who agreed that each expected them to use EBDM was higher for *all *programs compared to *chronic disease *programs (Table 3). In both categories, the highest percentage of respondents agreed that health department leaders expect them to use EBDM and the lowest percentage was among co-workers. Mississippi participants indicated that their overall top choices of incentive for using EBDM were: trainings and leaders in their organization placing a high priority on EBDM (Table 4). Those in the state office were more likely to prefer high priority among leadership.

**Table 3:** Expectations to use evidence-based decision making (n = 72), Mississippi, USA, 2010

	Total	State Office	District Office	p*
All Programs				

Health Dept. Leaders	75%	74%	77%	.78

Direct Supervisor	60%	66%	53%	.30

Community Partners	59%	55%	63%	.50

Co-workers	40%	45%	33%	.34

Chronic Disease Programs				

Health Dept. Leaders	65%	68%	60%	.47

Direct Supervisor	50%	61%	37%	.05

Community Partners	52%	55%	47%	.48

Co-workers	29%	37%	20%	.13

*p value for Pearson chi-square testing differences between state and district offices

**Table 4:** Incentives ranked as 1^st ^and within top 2 choices for using EBDM, Mississippi, USA 2010

	Total n = 68		State Office n = 38		District Office n = 30	
	**1^st ^Choice**	**Top 2**	**1^st ^Choice**	**Top 2**	**1^st ^Choice**	**Top 2**

**Trainings**	28%	57%	24%	58%	33%	57%

**EBDM High Priority**	37%	53%	45%	58%	27%	47%

**Positive Feedback**	13%	49%	11%	45%	17%	53%

**Performance Evaluation**	15%	29%	13%	26%	17%	33%

**Professional Recognition**	7%	12%	8%	13%	7%	10%

The highest rated skill in both surveys was the ability to find data (Kansas mean 7.4, 95%CI 7.1-7.8; Mississippi mean 8.0, 95%CI 7.4-8.5). The ability to use data for public health programming, grant writing or community assessment followed (Kansas mean 7.0, 95%CI 6.7-7.4; Mississippi mean 7.3, 95%CI 6.7-7.9) with developing evidence-based chronic disease programs as the lowest rated skill (Kansas mean 6.3, 95%CI 6.0-6.6; Mississippi mean 6.6, 95%CI 6.2-7.1).

Estimates of the percentage of evidence-based programs among all respondents from health departments were similar between Kansas and Mississippi. Kansas health department employees' mean estimate of the percentage of evidence-based programs in their agency was 65% (95%CI 61-70%). Mississippi survey respondents' overall mean estimate was 67% (95%CI 60-73%). Median estimates for both Kansas and Mississippi were 75%.

## Discussion

Despite increasing calls internationally for the inclusion of EBDM processes in public health programming, policymaking, and strategic planning [6-10,36-41], there is relatively sparse research to assess the workforce's current capacity at the local level. One notable exception is a needs assessments of population health staff conducted in New South Wales, Australia [42,43]. Their studies identified needs for technical support, training, and skills development, particularly among practitioners without master's degrees. The majority (55%) of practitioners recognized the need to increase their own capacity for EBDM. Practitioners indicated that their managers had more positive views than their own on the current promotion of evidence-based practice in population health while colleagues' views were less positive than their own. Based on this needs assessment, a working group identified evidence-based practice competencies [44]. Additionally, recent U.S. public health systems research seeks to better understand the variability in the quality and availability of public health services and to identify approaches that will improve service delivery, including the increased use of EBDM in agency settings [28-30].

As part of our study to increase EBDM capacity in U.S. public health agencies, we developed tailored survey tools to assess baseline EBDM capacity in two U.S. states. The core of our surveys was adopted from a previously testing instrument [22-24] and focused on practitioners' assessments of competencies previously determined to be critical to EBDM in chronic disease [33]. The practice of EBDM requires a broad skill set that includes the analysis and synthesis of evidence, quantitative and qualitative community assessments, and the use of program-planning frameworks [6]. The public health workforce is transdisciplinary by nature, and many who work in the field have no formal training in public health [45-47]. State-level practitioners in phase 1 of our study indicated that a lack of training necessary to conduct EBDM existed among both staff and managers [22]. Continued workforce training and capacity building is necessary, and the use of competencies to guide those efforts is critical for defining educational goals and outcomes [47,48].

Identification of the largest gaps in EBDM competencies within a state or locality provides health department leaders with actionable targets for the improvement of EBDM capacity. The three largest competency gaps in the Kansas and Mississippi surveys were consistent with findings from our national survey of state-level chronic disease practitioners [24] and may translate to other states. Our research shows that practitioners identify important targets for improving EBDM as: 1) communication with policymakers, 2) use of economic evaluation, and 3) translation of research to practice. These are skills that practitioners identify as important, yet unavailable, and such skills can be improved through trainings and technical support [49]. Because all of the competencies included on the survey are considered high or medium priority from previous research [33], agencies may also want to provide trainings for those competencies with low availability scores or may consider the use of incentives or priority-setting to improve competencies with low importance scores. Evidence-based public health trainings, based on these key EBDM competencies, have been found to be effective methods of integrating new knowledge and skills into the public health workforce [49-51]. As part of our research project, EBDM training courses were conducted in both Kansas and Mississippi to address gaps in competencies. While not attempted in our project, the use of knowledge brokers in Canada is another emerging and promising strategy for facilitating the translation of research to practice [52].

Practitioners in our surveys estimated that approximately two-thirds of programs in their agency were evidence-based. Mean estimates from Kansas (65%) and Mississippi (67%) were consistent with the 58% and 65% estimates obtained in follow-up surveys of EBDM training courses offered to public health professionals in Missouri and nationwide [49, unpublished data, Brownson]. Survey respondents were provided with a standard definition of EBDM before answering this question, but the results should still be interpreted with caution given they are self-reported and not objectively validated. In our qualitative results from phase 1 of this study, chronic disease practitioners identified a lack of consensus among practitioners regarding the precise meaning of the term 'evidence-based' as a barrier to the practice of EBDM [22]. The same program may be deemed 'evidence-based' by one practitioner and not another, and more objective measures are needed. A next logical step in this work is to compare self-reported data (e.g., on use of evidence-based interventions) with program reports (e.g., content analysis of grant applications).

New questions on the Mississippi survey provided results worthy of inclusion in subsequent surveys. Although sample sizes were relatively small, the expectation to use EBDM was lower for chronic disease programs compared to all programs, and the expectation from health department leaders nearly doubled that of co-workers. Creating a culture of EBDM in chronic disease control and prevention that encompasses all job types and levels of management will be an important step in increasing the use of EBIs [6]. Practitioners in our nationwide survey identified a lack of incentives for using EBDM as the highest of nine quantitatively measured barriers [23], and the Mississippi survey explored preferences for a range of incentives. Among Mississippi's customized list of incentives, respondents preferred leaders placing a "high priority" on EBDM and the provision of EBDM trainings. Leadership buy-in is a critical first step in order for practitioners to be able to utilize the knowledge and skills gained from EBDM trainings.

This survey's biggest limitation was that data were self-reported. We cannot directly validate our findings against a gold standard. Furthermore, the response rate in both states was low, and non-response bias is possible. Nearly half (51%) of Kansas recipients (a more diverse sample including community partners) and 25% of Mississippi recipients did not complete the survey. People with strong opinions on EBDM, either positive or negative, may have been more likely to respond. Data were not available to compare respondents with non-respondents across demographic characteristics. While this survey was created with ease of replication in mind, agencies with limited funds will not be able to offer gift cards incentives to increase response rates. Incentives for survey completion can take many forms, and agencies should use available resources.

## Conclusions

Top competency gaps in Kansas and Mississippi reinforced findings from our previous nationwide survey [24], indicating that, overall, practitioners need more training and tools for transmitting research to policymakers, making decisions based on economic evaluations, and translating EBIs to "real world" settings. Using our survey tool, health departments and NGOs can assess the unique EBDM capacity within their own workforce and use the localized survey findings to identify specific action points that will strengthen their EBDM capacity. These can include training programs focused on specific EBDM skills or can focus on incentives and policies that could affect the organizational culture and climate in a workplace [53]. EBDM is being advocated in many countries and by many health organizations. Our survey methods should be useful across numerous parts of the globe for assessing EBDM capacity and identifying approaches that will enhance the EBDM processes in public health programming and policymaking.

## Competing interests

The authors declare that they have no competing interests.

## Authors' contributions

Study concept and design: EAB, ADD, RCB. Development of survey tool and data interpretation: all authors. Data collection: JAJ, SW. Manuscript drafting: JAJ. Study supervision: RCB. All authors read and approved the final manuscript.

## Pre-publication history

The pre-publication history for this paper can be accessed here:

http://www.biomedcentral.com/1472-6963/12/57/prepub

## Supplementary Material

Additional file 1 **Survey instrument used in Kansas**. Click here for file

Additional file 2 **Survey instrument used in Mississippi**. Click here for file
