# Supplementary material for: A survey tool for measuring evidence-based decision making capacity in public health agencies
Source: BMC Health Serv Res. 2012 Mar 9;12:57. doi: 10.1186/1472-6963-12-57 (PMC3364859; doi:10.1186/1472-6963-12-57)
Supplement: Additional file 1 — Survey instrument used in Kansas. [file 1472-6963-12-57-S1.PDF]

BIOGRAPHICAL

Please answer the following questions about your background.

1. How do you best describe your agency/organization?
   ○ State Health Department
   ○ City or County Health Department
   ○ Coalition
   ○ Advocacy Group
   ○ University
   ○ Community-Based Organization
   ○ Voluntary Health Organization
   ○ Other (please specify)

2. Though you may work in several capacities, which do you consider your primary position? (**Required**)
   ○ Program Manager/Administrator/Coordinator
   ○ Health Educator
   ○ Epidemiologist
   ○ Statistician
   ○ Program Planner
   ○ Program Evaluator
   ○ Division or Bureau Head/Division Deputy Director
   ○ Department Head
   ○ Community Health Nurse, Social Worker, Dietitian, Nutritionist
   ○ Lay Health Worker
   ○ Other (please specify)

3. How long have you worked for this agency/organization?

   _____ Years
   _____ Months

4. How long have you been in your current position?

   _____ Years
   _____ Months

5. How long have you been involved in public health overall?

   _____ Years
   _____ Months

6. Do you specialize in a single program area or do you manage multiple areas? (**Required**)
○ I specialize in a single program area
○ I manage multiple program areas

7. In what single program area do you specialize? (**Required**)
○ Diabetes
○ Obesity
○ Physical Activity
○ Diet/Nutrition
○ Cancer Prevention and Control
○ Tobacco
○ Cardiovascular Health
○ Arthritis
○ Asthma
○ School Health
○ Women's Health, including Maternal and Child Health
○ Vision Preservation
○ Healthy Aging
○ Osteoporosis
○ Other (please specify)

8. In what other program areas do you specialize? (Check all that apply) (**Required**)
□ Diabetes
□ Obesity
□ Physical Activity
□ Diet/Nutrition
□ Cancer Prevention and Control
□ Tobacco
□ Cardiovascular Health
□ Arthritis
□ Asthma
□ School Health
□ Women's Health, including Maternal and Child Health
□ Vision Preservation
□ Healthy Aging
□ Osteoporosis
□ Other (please specify)

9. Please indicate the size of the population your agency serves.
   ○ 0 – 24,999
   ○ 25,000 – 49,999
   ○ 50,000 – 74,999
   ○ 75,000 – 99,999
   ○ 100,000 – 149,999
   ○ 150,000 – 199,999
   ○ 200,000 – 299,999
   ○ 300,000 – 399,999
   ○ 400,000+

10. Please indicate your gender.
   ○ Male
   ○ Female

11. What is the most advanced degree you have completed?
   ☐ High school or GED equivalent
   ☐ Some college
   ☐ Bachelor's degree
   ☐ BSN/RN/LPN
   ☐ MPH or MSPH
   ☐ MS, MA, or other masters degree
   ☐ DrPH
   ☐ PhD
   ☐ MD
   ☐ Other (please specify)

EVIDENCE-BASED DECISION MAKING

The next set of questions asks about evidence-based decision making. For the purposes of this survey, we think that evidence-based decision making involves the development, implementation, and evaluation of effective programs and policies in public health through:

- The systematic use of data and information systems
- The application of principles of scientific reasoning coupled with community engagement
- The appropriate use of behavioral science theory and program planning models

12. Based on your experience and best judgment, what percentage of programs in your agency is evidence-based?

   ____

13. What are some of the barriers to using evidence-based interventions or making evidence-based decisions that you have encountered?

14. In your opinion, what is the one thing that most needs to happen in your agency to increase the use of evidence-based decision making?

Please indicate the extent to which you agree or disagree with the following statement. Use the scale below to respond to each item by filling in the circle that best represents your response.

15. I feel that I have the skills necessary for developing evidence-based chronic disease control programs.

**Strongly Disagree**										**Strongly Agree**
**0**	**1**	**2**	**3**	**4**	**5**	**6**	**7**	**8**	**9**	**10**
○	○	○	○	○	○	○	○	○	○	○

IMPORTANCE AND AVAILABILITY OF EVIDENCE-BASED DECISION MAKING

Input from practitioners has led to the following definitions of skills needed to make evidence-based decisions to prevent chronic disease.

First, please use the blue scale to rate the importance of each skill.

Second, use the orange scale to rate how available you feel each skill is to you when you need it (either in your own skill set or in others').

16. Prioritizing Health Issues

Understand how to choose and implement appropriate criteria and processes for prioritizing program and policy options for chronic disease prevention.

	**Very Unimportant or Unavailable**										**Very Important or Available**
	**0**	**1**	**2**	**3**	**4**	**5**	**6**	**7**	**8**	**9**	**10**
**Level of importance**	○	○	○	○	○	○	○	○	○	○	○
**Current level of availability**	○	○	○	○	○	○	○	○	○	○	○

17. Evaluation Designs

Understand the various designs useful in chronic disease program evaluation with a particular focus on quasi-experimental, non-randomized designs.

	**Very Unimportant or Unavailable**										**Very Important or Available**
	**0**	**1**	**2**	**3**	**4**	**5**	**6**	**7**	**8**	**9**	**10**
**Level of importance**	○	○	○	○	○	○	○	○	○	○	○
**Current level of availability**	○	○	○	○	○	○	○	○	○	○	○

18. Qualitative evaluation (this type of evaluation uses non-numerical observations, using approved methods such as participant observation, group interviews, or focus groups)

Understand the importance of qualitative evaluation approaches including the steps involved in conducting them.

	**Very Unimportant or Unavailable**										**Very Important or Available**
	**0**	**1**	**2**	**3**	**4**	**5**	**6**	**7**	**8**	**9**	**10**
**Level of importance**	○	○	○	○	○	○	○	○	○	○	○
**Current level of availability**	○	○	○	○	○	○	○	○	○	○	○

19. Quantitative Evaluation (evaluation that uses data in numerical quantities, such as continuous measurements or counts)

Understand the value of quantitative evaluation approaches including the concepts of measurement validity and reliability.

18. Qualitative evaluation (this type of evaluation uses non-numerical observations, using approved methods such as participant observation, group interviews, or focus groups)

Understand the importance of qualitative approaches to chronic disease program evaluation.

	**Very Unimportant or Unavailable**										**Very Important or Available**
	**0**	**1**	**2**	**3**	**4**	**5**	**6**	**7**	**8**	**9**	**10**
**Level of importance**	○	○	○	○	○	○	○	○	○	○	○
**Current level of availability**	○	○	○	○	○	○	○	○	○	○	○

20. Developing an Action Plan for Program/Policy

Understand the importance of developing a chronic disease plan of action, which describes how the goals and objectives will be achieved, what resources are required, and how responsibility for achieving objectives will be assigned.

	Very Unimportant or Unavailable									Very Important or Available
	**0**	**1**	**2**	**3**	**4**	**5**	**6**	**7**	**8**	**9**	**10**
**Level of importance**	○	○	○	○	○	○	○	○	○	○	○
**Current level of availability**	○	○	○	○	○	○	○	○	○	○	○

21. Translating Evidence-Based Interventions

Understand the importance of translating evidence-based interventions to prevent chronic disease in unique "real world" settings.

	Very Unimportant or Unavailable									Very Important or Available
	**0**	**1**	**2**	**3**	**4**	**5**	**6**	**7**	**8**	**9**	**10**
**Level of importance**	○	○	○	○	○	○	○	○	○	○	○
**Current level of availability**	○	○	○	○	○	○	○	○	○	○	○

22. Transmitting Research to Policy Makers

Understand the importance of using creative ways of transmitting what is known to work (evidence-based interventions for chronic disease prevention) to policy makers in order to gain interest, political support and/or funding.

	Very Unimportant or Unavailable									Very Important or Available
	**0**	**1**	**2**	**3**	**4**	**5**	**6**	**7**	**8**	**9**	**10**
**Level of importance**	○	○	○	○	○	○	○	○	○	○	○
**Current level of availability**	○	○	○	○	○	○	○	○	○	○	○

23. Making Decisions Based on Economic Evaluation

Understand the importance of using economic data and strategies to evaluate costs and outcomes when making public health decisions related to chronic disease prevention.

	Very Unimportant or Unavailable										Very Important or Available
	**0**	**1**	**2**	**3**	**4**	**5**	**6**	**7**	**8**	**9**	**10**
**Level of importance**	○	○	○	○	○	○	○	○	○	○	○
**Current level of availability**	○	○	○	○	○	○	○	○	○	○	○

24. Multidisciplinary Partnerships

Understand the importance of developing traditional and non-traditional partnerships to address chronic disease with evidence-based strategies.

	Very Unimportant or Unavailable										Very Important or Available
	**0**	**1**	**2**	**3**	**4**	**5**	**6**	**7**	**8**	**9**	**10**
**Level of importance**	○	○	○	○	○	○	○	○	○	○	○
**Current level of availability**	○	○	○	○	○	○	○	○	○	○	○

The next questions ask about the use of data in public health assessment, program planning, and evaluation. We are referring to data sets like BRFSS, YRBSS, or CDC WONDER.

25. I am confident in my ability to ***find*** public health data and statistics on chronic disease risk factors.

**Strongly Disagree**										**Strongly Agree**
**0**	**1**	**2**	**3**	**4**	**5**	**6**	**7**	**8**	**9**	**10**
○	○	○	○	○	○	○	○	○	○	○

26. I am confident in my ability to ***use*** data and statistics for public health program planning, grant writing, and community assessment.

**Strongly Disagree**										**Strongly Agree**
**0**	**1**	**2**	**3**	**4**	**5**	**6**	**7**	**8**	**9**	**10**
○	○	○	○	○	○	○	○	○	○	○

POTENTIAL RESOURCES FOR EVIDENCE-BASED DECISION MAKING

If the following resources were easily available to help with the adoption of evidence-based decision making, how likely would you be to use each of them?

Use the scale below to respond to each item by filling in the circle that best represents your response.

27. Policy briefs combining statistical and narrative communication to provide information to policy makers about the importance of funding for evidence-based chronic disease control efforts.

**Least likely to use**										**Most likely to use**
**0**	**1**	**2**	**3**	**4**	**5**	**6**	**7**	**8**	**9**	**10**
○	○	○	○	○	○	○	○	○	○	○

28. Targeted workshops that would address significant barriers to using evidence-based interventions (e.g., economic evaluation, quantitative skills, translating evidence-based interventions).

**Least likely to use**										**Most likely to use**
**0**	**1**	**2**	**3**	**4**	**5**	**6**	**7**	**8**	**9**	**10**
○	○	○	○	○	○	○	○	○	○	○

29. Individualized technical consultation that would address barriers to using evidence-based interventions (e.g., one-on-one phone consultation).

**Least likely to use**										**Most likely to use**
**0**	**1**	**2**	**3**	**4**	**5**	**6**	**7**	**8**	**9**	**10**
○	○	○	○	○	○	○	○	○	○	○

30. Distance training opportunities via Webinar or video conference.

**Least likely to use**										**Most likely to use**
**0**	**1**	**2**	**3**	**4**	**5**	**6**	**7**	**8**	**9**	**10**
○	○	○	○	○	○	○	○	○	○	○

31. Distance training opportunities via conference call or CD-ROM.

**Least likely to use**										**Most likely to use**
**0**	**1**	**2**	**3**	**4**	**5**	**6**	**7**	**8**	**9**	**10**
○	○	○	○	○	○	○	○	○	○	○

32. A peer-to-peer network where you could discuss issues and ideas with colleagues.

**Least likely to use**										**Most likely to use**
**0**	**1**	**2**	**3**	**4**	**5**	**6**	**7**	**8**	**9**	**10**
○	○	○	○	○	○	○	○	○	○	○

33. Are there other resources that you would find helpful in using evidence-based decision making? What are they?
